# Study of immune reconstitution inflammatory syndrome during antiretroviral therapy in South Indian HIV patients

**DOI:** 10.1186/1471-2334-14-S3-P53

**Published:** 2014-05-27

**Authors:** A Pramod Kumar, G Parthasarathi, SN Mothi, VHT Swamy, AP Sudheer, S Rajendra Prasad

**Affiliations:** 1 JSS College of Pharmacy, Department of Clinical Pharmacy, JSS University, Mysore, India; 2 Asha kirana Hospital; Center for AIDS care and Research, Mysore, India; 3 Vivekananda Memorial Hospital, Saragur, Mysore, India

## Background

Immune reconstitution inflammatory syndrome (IRIS) is a common cause of death and even more common cause of morbidity in patients started on highly active antiretroviral therapy (HAART). At present, very limited data is available about IRIS in the local population so the present study was conducted to determine the incidence, clinical manifestations, risk factors and outcomes of IRIS in south Indian HIV/AIDS patients.

## Methods

Study was a prospective surveillance method to identify IRIS from two ART centers in Mysore city, India for a period of two years. HIV patients who are initiated with HAART were included for the study and were followed up for a period of six months. Data were analyzed using SPSS version 21.

## Results

Out of 798 people followed 82 patients had experienced IRIS with an incidence rate of 10%. Among them 48% were unmasking and rest were paradoxical worsening. Diagnosis included tuberculosis (44%), herpes zoster (18%), cryptococcal meningiti*s* (11%) and other opportunistic infections (27%). The major manifestation of IRIS were fever (44%) followed by lymphadenitis (36%). Statistically important predictors for occurrence of IRIS were male gender and low CD4 count. Two deaths were attributable to IRIS and 54% of them required hospitalization.

## Conclusion

Patients with advanced immunodeficiency at HAART initiation are at greatest risk of developing IRIS and should be appropriately screened and monitored. Educational programs on recognizing and treating these conditions should be initiated in ART access programs.

